# Domains of disgust sensitivity: revisited factor structure of the questionnaire for the assessment of disgust sensitivity (QADS) in a cross-sectional, representative german survey

**DOI:** 10.1186/1471-2288-10-95

**Published:** 2010-10-20

**Authors:** Katja Petrowski, Sören Paul, Gabriele Schmutzer, Marcus Roth, Elmar Brähler, Cornelia Albani

**Affiliations:** 1 Dresden University of Technology, Department of Psychotherapy and Psychosomatic Medicine, Fetscherstrasse 74, D-01307 Dresden, Germany; 2 University of Leipzig, Department of Medical Psychology and Medical Sociology, Philipp-Rosenthal-Strasse 55, D-04103 Leipzig, Germany; 3 University of Leipzig, Department of Psychology II, Seeburgstrasse 14-20, D-04103 Leipzig, Germany

## Abstract

**Background:**

Disgust sensitivity is defined as a predisposition to experiencing disgust, which can be measured on the basis of the Disgust Scale and its German version, the Questionnaire for the Assessment of Disgust Sensitivity (QADS). In various studies, different factor structures were reported for either instrument. The differences may most likely be due to the selected factor analysis estimation methods and the small non-representative samples. Consequently, the aims of this study were to explore and confirm a theory-driven and statistically coherent QADS factor structure in a large representative sample and to present its standard values.

**Methods:**

The QADS was answered by N = 2473 healthy subjects. The respective households and participants were selected using the random-route sampling method. Afterwards, the collected sample was compared to the information from the Federal Statistical Office to ensure that it was representative for the German residential population. With these data, an exploratory Promax-rotated Principal Axis Factor Analysis as well as comparative confirmatory factor analyses with robust Maximum Likelihood estimations were computed. Any possible socio-demographic influences were quantified as effect sizes.

**Results:**

The data-driven and theoretically sound solution with the three highly interrelated factors Animal Reminder Disgust, Core Disgust, and Contamination Disgust led to a moderate model fit. All QADS scales had very good reliabilities (Cronbach's alpha) from .90 to .95. There were no age-differences found among the participants, however, the female participants showed remarkably higher disgust ratings.

**Conclusions:**

Based on the representative sample, the QADS factor structure was revised. Gender-specific standard percentages permit a population-based assessment of individual disgust sensitivity. The differences of the original QADS, the new solution, and the Disgust Scale - Revised will be discussed.

## Background

Disgust sensitivity describes an individual's time-invariant, genetically-based personality trait, a predisposition to reacting to specific materials with disgust [[1], p.111; [2]]. The specific materials that trigger disgust can be grouped into five theoretically proposed categories of disgust elicitors [3]: badly tasting substances can produce *Distaste*, which protects the body from poisons. *Core Disgust *can be provoked by rotten food, body products, rodents, and other small vermin in order to protect the body from disease or infection. *Animal Nature/Reminder Disgust *refers to sex, death, poor hygiene, and body envelope violations for protection against death and mortality. *Interpersonal-Contamination Disgust *protects the body by limiting the contact with strangers and other undesirables. *Moral Disgust *mainly protects the social order in case of moral offenses such as rape or murder.

Based on the 32 items of the original *Disgust Scale (DS) *by Haidt, McCauley, and Rozin [4] only three of the eight DS factors were found to be psychometrically stable. These three stable factors are Core Disgust, Animal Reminder Disgust, and Contamination Disgust with 25 items overall which represent the *Disgust Scale - Revised (DS-R) *by Olatunji and colleagues [5]. The reliabilities of the DS-R scales varied from (Cronbach's Alpha) .71 to .82.

Schienle, Walter, Stark, and Vaitl [1] translated all the items from the original DS into German and implemented a consistent five-point rating scale. Unfortunately, the translation led to insufficient psychometric properties. Therefore, 28 newly generated expert-rated items - four items for each of the existing DS scales and four items for a new scale called Deformation - were included. In a second step, any item of the translated and extended DS whose Measurement of Sampling Adequacy came to > .70, was excluded. In a third step, the factor structure was tested.

The parallel analysis and an obliquely rotated Principal Axis factor analysis revealed five factors: Death/Deformation, Body Secretions, Spoilage, Poor Hygiene, and Oral Rejection. In order to adjust the unsatisfactory internal consistency of .66 of Poor Hygiene, four more items were generated. The following confirmatory factor analysis of the 39-item-version revealed a Root Mean Square Error of Approximation (RMSEA) of .06 which suggests a sufficient model fit. Two more items were excluded as their factor loadings were < .30. The final German instrument "Fragebogen zur Ekelempfindlichkeit" will be referred to as "Questionnaire for the Assessment of Disgust Sensitivity" (QADS) in the remainder of this article and can be found as Additional file 1. Even though modest to good reliabilities were reported for all the scales (.69 to .85), the calculations of the final version were based on a small non-representative sample of N = 220 participants.

The discrepancies in the factor structures of the QADS and the DS/DS-R as well as the following shortcomings led to the necessity of revisiting and possibly refining the QADS factor structure.

Most of the studies on the DS factor structure applied estimation methods that did not take into account the non-normality of item distributions [5]. The exact estimation method of the QADS' confirmatory factor analysis had not been reported by Schienle et al. [1], therefore, the validity of that factor structure is questionable at best. Furthermore, the QADS factor structure was calculated based on a small, non-representative sample and had not yet been replicated. From a theoretical point of view, the scales do not clearly represent or directly refer to the groups of disgust sensitivity elicitors as proposed by Rozin, Haidt, and McCauley [3].

Consequently, the first aim of this study was to find and confirm a new, statistically coherent, data-driven and theoretically sound factor structure on a large representative German sample while applying adequate estimation methods. The second aim was to compare the fit of this structure to the fit of alternative structures in the present sample. Third, the standard percentages will be accounted for.

## Methods

### Sample

The data collection was conducted on behalf of the University of Leipzig and obtained in the fall of 2004 from the USUMA Berlin polling institute within a representative, multi-topic survey. The participants were interviewed at their homes. The households and the participants were selected by the random-route sampling method which ensures that the sample resembles the population in its relevant characteristics [6]. This sample was then compared with the information from the Federal Statistical Office in order to obtain a truly representative sample of the German residential population. The coverage rate was 62.3% with 2591 participants aged 14 to 99. Of these, the number of N = 2473 who were native German speakers were examined. Further details concerning the individual socio-demographic properties can be found in Table 1.

**Table 1:** Socio-demographic sample characteristics

		All (N = 2473)	
**Gender**	male	1171	47.4%
	female	1302	52.6%

**Age **(years)	mean	48.14	
	standard deviation	17.97	
	range	14-99	

**Age groups** (years; N = 2349)	< 25	262	11.1%
	25 - 34	331	14.1%
	35 - 44	479	20.4%
	45 - 54	383	16.3%
	55 - 64	381	16.2%
	65 - 74	344	14.7%
	> 74	169	7.2%

**Marital status**	married, living together	1299	52.5%
	married, living separately	26	1.1%
	single	587	23.7%
	divorced	258	10.4%
	widowed	303	12.3%

**Education**	not graduated	36	1.5%
	pupil	67	2.7%
	8^th ^grade (Hauptschule)	1112	45.0%
	10^th ^grade (Mittlere Reife/Realschule/POS)	840	34.0%
	technical school	70	2.8%
	12^th^/13^th ^grade (Abitur)	177	7.2%
	university/college degree	171	6.9%

**Employment status**	full-time (≥ 35 hours)	899	36.4%
	part-time (15-34 hours)	189	7.6%
	part-time (≤ 14 hours)	49	2.0%
	community service/parental leave	37	1.5%
	unemployed	168	6.8%
	pensioner	754	30.5%
	unable to work	185	7.5%
	in professional training	35	1.4%
	in school-/college education	157	6.4%

**Household income** (net)	< 750 € per month	105	4.5%
	750 to 1250 € per month	507	21.5%
	1250 to 2000 € per month	911	38.6%
	> 2000 € per month	838	35.5%

All the participants volunteered and received a data protection declaration in agreement with the Helsinki Declaration. The study was approved according to the ethical guidelines of the "German Professional Institutions for Social Research" [Arbeitskreis Deutscher Markt- und Sozialforschungsinstitute, Arbeitsgemeinschaft Sozialwissenschaftlicher Institute, Berufsverband Deutscher Markt- und Sozialforscher].

### Instrument

The *Questionnaire for the Assessment of Disgust Sensitivity, QADS *[1] consists of 37 items rated on a five-point Likert scale (0 = not disgusting, 4 = very disgusting) with six to nine items per scale to be rated on how disgusting a statement is, e.g. "you try to eat monkey meat", "you touch a dead body", or "you are about to drink a glass of milk when you smell that it's spoilage". Internal consistencies of the five scales came to: Death/Deformation = .85, Body Secretion = .74, Poor Hygiene = .78, Spoilage = .72 and Oral Rejection = .69 [1].

### Statistical Analysis

First, an item analysis was computed to test for the non-normality of the item distributions. Second, an exploratory factor analysis (EFA) was conducted to find a data-driven, statistically coherent, and theoretically sound solution factor structure. Third, a confirmatory factor analysis (CFA) tested the fit of the factor solution in a representative sample while multiple fit indices of several models were compared to find the most preferable solution. According to Olatunji et al. [5] and Brown [7], specific estimation and extraction methods had to be chosen in case of non-normality, i.e. Principal Axis extraction in the EFA [7] and robust Maximum Likelihood estimation in the CFA. Since conducting both, the EFA and the CFA, using the same sample would lead to an artificially increased model fit, the sample was randomly divided into two partial samples, one for each upcoming procedure (n_CFA _= 1252, n_EFA _= 1221). No significant differences of means were found between the two partial samples for all the QADS items and the socio-demographic variables with all t (1, 2550) < 1.45 (p_twosided _> .15).

Possible gender differences were calculated with Student t-tests. As Table 1 shows, age was divided into seven groups in order to compute a one-way analysis of variance and test for possible specific cohort effects. These effects might not be assessable through simple correlation analyses. Additionally, a subsequent t-test would be susceptible to biases caused by unequal cell sizes.

## Results

### Descriptive item analysis

This step was conducted using SPSS 16.0 as well as LISREL 8.80s/PRELIS 2.80s. Table 2 presents the item analyses for the QADS items. All 37 items were considered to be disgusting by at least 77% of the participants while the appraisal to 21 items was above the mean overall appraisal (M = 2.50). Consistent with the findings from the DS [5], most of the item distributions demonstrated significant univariate skewness and kurtosis as well as significant univariate non-normality in the Shapiro-Wilk test of normality [8] with W > .77 (p < .01). Further, significant multivariate non-normality was found with Mardia's multivariate skew (β_1, p _= 83.9, χ^2 ^= 33417.6, p = .000) and Mardia's multivariate kurtosis (β_2, p _= 1804.4, N (β_2, p_) = 164.4, p = .000) [9]. Therefore, appropriate estimation procedures should be applied in the EFA [5,7].

**Table 2:** QADS item characteristics (N = 2349 to 2457)

Item	M (SD)	skewness	kurtosis	W (N)	% 0	% 1	% 2	% 3	% 4
01 Someone doesn't clean his/her hands after using the restroom. ^4^	2.5 (1.2)	-0.42**	-0.65**	.89**	5	13	28	29	25
02 You are biting into a grilled grasshopper. ^5^	3.0 (1.2)	-0.95**	-0.11	.80**	4	9	17	22	48
03 You smell vomit. ^5^	3.2 (1.0)	-1.20**	0.83**	.77**	2	6	13	29	50
04 You have to remove a hairy dead spider from your room. ^3^	2.2 (1.4)	-0.19**	-1.26**	.88**	15	18	21	20	26
05 Someone profusely smelling of sweat takes the bus seat next to you. ^2^	2.9 (1.0)	-0.66**	-0.23**	.86**	2	8	23	34	33
06 You enter a crypt, where there are coffins. ^1^	2.1 (1.4)	-0.13**	-1.18**	.90**	16	18	24	21	21
07 You are eating a steak and find that it is still rare on the inside. ^5^	2.1 (1.4)	-0.07	-1.20**	.90**	17	18	25	19	21
08 You try to eat monkey meat. ^5^	3.1 (1.2)	-1.07**	0.15**	.78**	4	7	17	21	51
09 A friend tells you he generally doesn't use a deodorant. ^4^	1.8 (1.3)	0.15**	-1.05**	.91**	20	22	27	18	13
10 You see a cockroach in someone's house. ^3^	2.4 (1.3)	-0.31**	-0.89**	.90**	9	16	27	25	23
11 You hear the mucus rattle as someone is clear- ing his/her throat. ^2^	2.7 (1.1)	-0.55**	-0.47**	.88**	5	10	26	30	29
12 You see someone vomit. ^5^	3.0 (1.0)	-0.96**	0.35**	.82**	3	6	18	31	42
13 You touch a dead body. ^1^	2.4 (1.3)	-0.34**	-0.95**	.89**	12	13	27	22	26
14 You accidentally touch the toilet seat in a public restroom. ^4^	2.6 (1.2)	-0.49**	-0.63**	.88**	7	11	27	28	27
15 You visit your favorite restaurant, and the cook has a cold. ^4^	2.0 (1.2)	-0.02	-0.92**	.91**	14	20	31	21	14
16 You are to ride in a hearse. ^1^	2.0 (1.4)	-0.06	-1.14**	.90**	18	17	26	21	18
17 While eating soup, your tongue comes in contact with a piece of hair. ^2^	2.7 (1.1)	-0.51**	-0.61**	.89**	4	12	25	29	30
18 You smell spoiled food. ^3^	2.9 (1.1)	-0.77**	-0.09	.85**	3	8	21	34	34
19 Someone with dirty fingernails hands you a book. ^4^	2.1 (1.2)	-0.14**	-0.78**	.91**	11	18	33	24	14
20 During a walk in the woods, you see a decomposing animal. ^3^	2.2 (1.3)	-0.15**	-0.95**	.91**	13	18	28	24	17
21 While assisting in a medical emergency, you are to press against a heavily bleeding wound. ^1^	1.9 (1.3)	0.07**	-1.10**	.91**	21	20	26	20	13
22 A bad odor reaches your nose. You look down and see that you have stepped into dog feces. ^2^	2.8 (1.1)	-0.67**	-0.37	.86**	3	11	21	32	33
23 You enter a heavily soiled gas-station restroom. ^4^	3.3 (0.9)	-1.23**	0.92**	.75**	1	4	14	27	54
24 You touch a dead person's head. ^1^	2.5 (1.3)	-0.47**	-0.93**	.88**	11	13	22	24	30
25 Someone with terribly bad breath speaks to you. ^2^	3.0 (1.0)	-0.68**	-0.10**	.85**	1	7	21	38	33
26 You have touched the stump of someone's amputated limb. 1	2.0 (1.3)	0.02**	-1.07**	.91**	18	20	27	20	15
27 You see someone put ketchup on vanilla ice cream and eat it. ^3^	1.7 (1.3)	0.24	-1.02**	.90**	23	22	27	16	12
28 You are about to drink a glass of milk when you smell that it's spoiled. ^3^	2.5 (1.2)	-0.41**	-0.67**	.90**	6	14	27	30	23
29 You see maggots on a piece of meat in an outdoor garbage pail. ^3^	2.9 (1.1)	-0.91**	0.04**	.83**	4	8	18	30	40
30 You are walking barefoot on concrete and you step on an earthworm. ^3^	2.2 (1.3)	-0.16**	-1.12**	.90**	14	18	24	23	21
31 While you are walking through a tunnel under a railroad track, you smell urine. ^2^	2.8 (1.1)	-0.64**	-0.30**	.87**	3	10	23	34	30
32 You accidentally touch the ashes of a person who has been cremated. ^1^	2.3 (1.3)	-0.20**	-1.06**	.90**	13	17	26	21	23
33 You are hungry. In front of you there is a bowl of your favorite soup that had been stirred with a used but thoroughly washed flyswatter. ^4^	2.8 (1.2)	-0.66**	-0.48**	.86**	4	11	22	27	36
34 You see a person with greasy hair. ^4^	2.2 (1.1)	-0.13**	-0.72**	.91**	7	19	33	26	15
35 In a restaurant, you see someone eat his food messily with his fingers. ^2^	2.5 (1.2)	-0.44	-0.63**	.89**	6	13	27	30	24
36 You discover that a friend of yours changes his/her underwear only once a week. ^4^	2.7 (1.2)	-0.56	-0.57**	.88**	5	12	24	29	30
37 You take raw egg-white into your mouth. ^5^	2.5 (1.3)	-0.48**	-0.85**	.88**	10	12	24	25	29

Note. Item numbering according to Schienle et al. [01]. Original factors: ^1 ^"Death/Deformation", ^2 ^"Body Secretions", ^3 ^"Spoilage", ^4 ^"Poor Hygiene", ^5 ^"Oral Rejection". M Mean (Range 0 - 4, lower/higher scores = refusal/stronger approval). SD standard deviation. W = Shapiro-Wilk test of normality. df_W _= 2524. Univariate non-normality tests of skewness and kurtosis: ** p < .01. 0-4% = frequencies of answers (0 = not disgusting, 4 = very disgusting). Items 27-32 and 36 adopted from Olatunji et al. [05], items 8, 10-15, 33, 35 resemble the ones from Olatunji et al. [05], every other item was translated by the authors.

### Exploratory Factor Analysis (EFA)

The EFA (n_EFA _= 1221) was computed using the Principal Axis extraction method in SPSS 16.0 in order to adjust to non-normal item distributions [7]. A Parallel Analysis provided the most convincing argument for determining the number of factors to retain [10,11] and led to a four-factor-solution (EFA eigenvalues: 14.53, 2.57, 1.52, 1.39, 1.04; parallel analysis average eigenvalues: 1.34, 1.30, 1.27, 1.25, 1.23). As all the items tended to measure disgust sensitivity in related domains (i.e. disgust elicitors), the factors were obliquely rotated with Promax. The user-defined Promax power parameter Kappa was systematically altered between 1, 2, 4, 5 and 6 to find the most appropriate solution (see [12] pp.190-197). Exclusively, the items showing a substantial loading of > .30 on at least one factor were interpreted, i.e. non-hyperplane items (see [5], p.285; [7] p.130).

An EFA with four factors revealed one hyperplane item in several solutions (item 37) while Kappa = 5 led to the least number of complex items (items 5, 17, 27, 33, 36). In every one of the rotated factor solutions, at least two of the three items that had salient loadings on factor four, also loaded considerably on another factor. Factor four undermined the simplicity of a latent structure (see [13] p.335), was difficult to interpret, and accounted for less than five items, thus it was probably an obsolete factor. Furthermore, the literature on disgust sensitivity prefers three-factor-solutions whenever "moral disgust" is not to be considered [3,5].

Consequently, a similar EFA with three factors was computed. Kappa = 5 led to five complex items, but no hyperplane item. Table 3 presents factor loadings for the rotated three-factor solution including the complex items mentioned. The highly correlated factors (r_12 _= .60; r_13 _= .76; r_23 _= .71) accounted for 11.3% of the total variance for factor one, and 10.8% and 12.3% for factors two and three, respectively. Due to factor inter-correlations, these sums of squared factor loadings need not be added up.

**Table 3:** QADS communalities, factor loadings (principal axis analysis, Promax-rotated, Kappa = 5, N = 1221) and item-scale-correlations

Item	**h**^**2**^	Factor			ISC
		1	2	3	
**Factor 1 - Core Disgust**					
03 You smell vomit. ^5^	.47	**.84**	.01	-.24	.59
23 You enter a heavily soiled gas-station restroom. ^4^	.48	**.79**	-.22	.01	.60
*12 You see someone vomit. ^5^*	.*42*	***.73***	.*12*	-.*21*	.*57*
18 You smell spoiled food. ^3^	.51	**.66**	.13	-.04	.67
25 Someone with terribly bad breath speaks to you. ^2^	.41	**.60**	-.04	.09	.61
*29 You see maggots on a piece of meat in an outdoor garbage pail. ^3^*	.*48*	***.58***	.*14*	.*03*	.*64*
22 A bad odor reaches your nose. You look down and see that you have stepped into dog feces. ^2^	.38	**.56**	.01	.06	.56
*31 While you are walking through a tunnel under a railroad track, you smell urine. ^2^*	.*50*	***.53***	-.*04*	.*25*	.*64*
02 You are biting into a grilled grasshopper. ^5^	.31	**.44**	.07	.09	.52
05 Someone profusely smelling of sweat takes the bus seat next to you. ^2^	.49	**.43**	-.16	.43	.60
*08 You try to eat monkey meat. ^5^*	.*31*	***.38***	.*19*	.*05*	.*49*
*28 You are about to drink a glass of milk when you smell that it's spoiled. ^3^*	.*34*	***.37***	.*21*	.*07*	.*55*
*33 You are hungry. In front of you there is a bowl of your favorite soup that had been stirred with a used but thoroughly washed flyswatter. ^4^*	.*39*	***.35***	-.*03*	***.34***	.*58*
37 You take raw egg-white into your mouth. ^5^	29	**.33**	.12	.15	.51
17 While eating soup, your tongue comes in contact with a piece of hair. ^2^	.49	**.31**	.08	**.38**	.63

**Factor 2 - Animal Reminder Disgust**					
*13 You touch a dead body. ^1^*	.*60*	.*15*	***.87***	-.*29*	.*69*
16 You are to ride in a hearse. ^1^	.66	-.12	**.81**	.10	.74
24 You touch a dead person's head. ^1^	.59	.22	**.81**	-.26	.71
*32 You accidentally touch the ashes of a person who has been cremated. ^1^*	.*63*	.*00*	***.76***	.*05*	.*73*
26 You have touched the stump of someone's amputated limb. ^1^	.53	-.15	**.75**	.09	.67
21 While assisting in a medical emergency, you are to press against a heavily bleeding wound. ^1^	.44	.02	**.69**	-.06	.63
06 You enter a crypt, where there are coffins. ^1^	.55	-.10	**.68**	.16	.68
20 During a walk in the woods, you see a decomposing animal. ^3^	.51	.09	**.51**	.18	.65
*30 You are walking barefoot on concrete and you step on an earthworm. ^3^*	.*44*	.*12*	***.33***	.*29*	.*55*

**Factor 3 - Contamination Disgust**					
34 You see a person with greasy hair. ^4^	.51	-.05	-.17	**.86**	.64
19 Someone with dirty fingernails hands you a book. ^4^	.52	-.15	.17	**.70**	.66
09 A friend tells you he generally doesn't use a deodorant. ^4^	.47	.-23	.19	**.69**	.63
01 Someone doesn't clean his/her hands after using the restroom. ^4^	.46	.20	-.23	**.66**	.59
35 In a restaurant, you see someone eat his food messily with his fingers. ^2^	.47	.17	-.09	**.61**	.60
*15 You visit your favorite restaurant, and the cook has a cold. ^4^*	.*52*	-.*08*	.*22*	***.61***	.*66*
*10 You see a cockroach in someone's house. ^3^*	.*53*	.*07*	.*10*	***.60***	.*70*
*36 You discover that a friend of yours changes his/her underwear only once a week. ^4^*	.*47*	***.31***	-.*16*	***.53***	.*60*
04 You have to remove a hairy dead spider from your room. ^3^	.41	-.06	.22	**.51**	.58
*14 You accidentally touch the toilet seat in a public restroom. ^4^*	.*47*	.*24*	.*12*	***.40***	.*62*
*27 You see someone put ketchup on vanilla ice cream and eat it. ^3^*	.*34*	-.*12*	***.32***	***.40***	.*49*
*11 You hear the mucus rattle as someone is clearing his/her throat. ^2^*	.*34*	.*23*	.*00*	***.39***	.*54*
07 You are eating a steak and find that it is still rare on the inside. ^5^	.36	.02	.29	**.34**	.52

Note. h^2 ^communality. α Cronbach's Alpha for internal consistency. *Disgust Scale - Revised (DS-R) *items are printed in italics. Item numbering according to Schienle et al. [01]. Original factors: ^1 ^"Death/deformation", ^2 ^"Body secretions", ^3 ^"Spoilage", ^4 ^"Poor hygiene", ^5 ^"Oral rejection". ISC corrected item-scale-correlation. Items 27-32 and 36 adopted from Olatunji et al. [05], items 8, 10-15, 33, 35 resemble the ones from Olatunji et al. [05], every other item was translated by the authors.

The properties of the QADS factors can be seen in Table 4. The factor distributions did not tend to be significantly skewed, except for Animal Reminder Disgust (γ_1 _= -47). All the distributions were significantly flatter than the Gaussian distribution (γ_2 _= -.23 to -.71).

**Table 4:** QADS gender-specific descriptives (N = 2349)

QADS factor	Items	Observed Range	All	Male	Female	Reliabilities		Skewness	Kurtosis
			M (SD)	M (SD)	M (SD)	Split-Half	Cronbach's Alpha		
Main Score	37	17-148	93.5 (26.8)	86.5 (26.7)	99.6 (25.4)	.92	.95	-.02	-.49**
Animal Reminder Disgust	15	7-60	43.8 (10.4)	41.4 (10.6)	45.9 (9.7)	.88	.90	-.47**	-.23*
Core Disgust	9	1-36	19.8 (8.7)	18.0 (8.6)	21.4 (8.6)	.89	.90	-.09	-.71**
Contamination Disgust	13	2-52	30.0 (10.6)	27.2 (10.6)	32.4 (10.0)	.86	.90	.04	-.51**

Note. M Mean (lower/higher scores = refusal/stronger approval). SD standard deviation. Male N = 1098. Female N = 1251, split-half reliabilities adjusted after Spearman-Brown. Univariate non-normality tests of skewness and kurtosis: ** p < .01. * p < .05.

### Confirmatory Factor Analysis (CFA)

In order to test the proposed three-factor model and alternative models for their fit in the respective partial sample (n_CFA _= 1252), several CFAs were computed using Mplus 5.1. The input data consisted of interval-scaled raw values. Missing data was excluded listwise, resulting in n = 1206 examined cases. The robust Maximum Likelihood Method of estimation was used in order to account for the significant non-normality of the data [7]. The item assignments of the three-factor model can be seen in Table 2. In order to maximize the simplicity of the structure, no cross-loadings between items were allowed, and errors were specified as random and uncorrelated. Items 3, 13 and 34 were chosen to be marker indicators since these items had the highest salient loadings on their respective latent factors. The unrestricted one-factor model and the five-factor model proposed by Schienle and colleagues [1] constituted the alternative models.

For the three-factor model, all observed and standardized factor loadings were significant with p = .000. For factor one, loadings varied between .54 and .71 (standardized errors were .015 to .020), for factor two between .63 and .79 (.011 to .018), and for factor three between .52 and .74 (.014 to .020). The standardized residual variances of the items varied between .38 and .72. Similar to the EFA results, the three factors were highly and significantly intercorrelated (r_12 _= .71; r_13 _= .88; r_23 _= .80; all p = .000).

To evaluate model fits, the following thresholds appear to be appropriate. The χ^2^/df index should be < 2.0 as mentioned by Bollen [14]. According to recommendations by Hu and Bentler [15], the Comparative Fit Index (CFI) as well as the Tucker-Lewis-Index (TLI) must be above .95 and the Standardized Root Mean Square Residual (SRMR) under .08. Browne and Cudeck [16] found a RMSEA of less than .05 to be good and of .05 to .079 to be adequate. The Akaike Information Criterion (AIC) is used in the comparison of the model fit for those non-nested models (see [7], pp.175-181), giving preference to the model with the lowest AIC.

The overall fit statistics suggest poor fit for the one-factor solution, χ^2 ^(629) = 5,296.048, p < .001, SRMR = .068, RMSEA = .079, TLI = .736, CFI = .750, and moderate fit for both the three-factor model, χ^2 ^(626) = 3,989.355, p < .001, SRMR = .061, RMSEA = .067, TLI = .81, CFI = .82 and the five-factor model, χ^2 ^(619) = 6,967.598, p < .001, SRMR = .066, RMSEA = .055, TLI = .816, CFI = .829. The model comparison fit statistics revealed that the three-factor model (AIC = 122,571.362) has a slightly better fit than the one-factor model (AIC = 122,751.471) and definitely a better fit than the five-factor model (AIC = 243,078.898). Therefore, the three-factor model seems to be superior to the alternative models. Modifications to this preferred three-factor model, e.g. by reassigning several or all cross-loading items to other factors, or leaving these items out, were tested. However, at best, the fit indices resulted either in being inflated or in being equal.

### Socio-demographic influences

The stability of the three-factor-structure was examined within several groups of the sample (N = 2743) using SPSS 16.0. Table 4 shows that on all QADS factors the female participants reported a significantly higher disgust sensitivity with all t (1, 2348) > 9.6 (p < .001), producing significant medium-sized effects on the *Main Score *(Cohen's d = 0.50), *Core Disgust *(Cohen's d = 0.44), *Animal Reminder Disgust *(Cohen's d = 0.45) and *Contamination Disgust *(Cohen's d = 0.51).

The one-way analysis of variance for age differences yielded significant but meaningless age-effects on the factors such as all F (6, 2342) = 3.04 to 4.52 (p < .01) and all η^2^_part _< .02, making possible post-hoc tests unnecessary. No factor-specific patterns occurred for gender or age.

### Standard percentages of QADS

Since distributions tended to be non-Gaussian and no meaningful age specificities but gender specificities had been found for QADS factors, standard percentages are presented separately for male and female participants in Tables 5 and 6.

**Table 5:** QADS main score standard percentages for males (N = 1098) and females (N = 1251)

Raw value	Main Score		Raw value	Main Score		Raw value	Main Score	
	Male	Female		Male	Female		Male	Female
**17**	.1	--	**61**	***17.0***	6.4	**105**	***75.0***	***58.3***
**18**	.2	--	**62**	***17.7***	6.8	**106**	***75.9***	***60.0***
**19**	.2	--	**63**	***18.9***	7.2	**107**	***76.5***	***61.2***
**20**	.4	--	**64**	***19.8***	7.8	**108**	***77.1***	***62.5***
**21**	.4	--	**65**	***20.9***	8.3	**109**	***78.3***	***63.5***
**22**	.5	--	**66**	***22.0***	9.2	**110**	***79.1***	***64.7***
**23**	.5	--	**67**	***23.8***	10.0	**111**	***80.4***	***66.3***
**24**	.5	--	**68**	***25.0***	10.9	**112**	***81.3***	***67.9***
**25**	.6	--	**69**	***26.6***	11.4	**113**	***82.0***	***68.6***
**26**	1.0	.1	**70**	***27.4***	12.2	**114**	***83.1***	***69.9***
**27**	1.2	.2	**71**	***28.7***	13.0	**115**	***84.0***	***71.3***
**28**	1.4	.2	**72**	***30.0***	14.2	**116**	***85.2***	***72.2***
**29**	1.5	.2	**73**	***31.7***	15.4	**117**	85.8	***73.3***
**30**	1.8	.2	**74**	***34.2***	***16.5***	**118**	87.2	***74.3***
**31**	2.0	.2	**75**	***36.0***	***17.9***	**119**	87.9	***75.3***
**32**	2.1	.2	**76**	***37.5***	***19.1***	**120**	89.2	***76.7***
**33**	2.2	.3	**77**	***39.3***	***20.2***	**121**	89.8	***77.9***
**34**	2.6	.3	**78**	***40.9***	***22.2***	**122**	90.7	***79.0***
**35**	2.7	.5	**79**	***42.4***	***23.5***	**123**	91.3	***80.3***
**36**	2.8	.5	**80**	***44.3***	***24.8***	**124**	92.0	***81.0***
**37**	3.2	.6	**81**	***45.7***	***26.3***	**125**	92.2	***81.9***
**38**	3.8	.6	**82**	***46.4***	***27.4***	**126**	93.0	***83.2***
**39**	4.1	.7	**83**	***47.8***	***28.8***	**127**	93.3	***84.2***
**40**	4.2	.7	**84**	***49.6***	***30.1***	**128**	93.9	***84.8***
**41**	4.4	.8	**85**	***51.3***	***31.9***	**129**	94.2	85.7
**42**	4.9	1.0	**86**	***52.7***	***33.3***	**130**	94.6	86.2
**43**	5.3	1.1	**87**	***54.2***	***33.9***	**131**	94.9	87.1
**44**	5.8	1.3	**88**	***55.2***	***35.3***	**132**	95.2	88.3
**45**	5.9	1.4	**89**	***56.8***	***36.5***	**133**	95.6	89.2
**46**	6.3	1.4	**90**	***58.3***	***37.3***	**134**	95.9	89.8
**47**	6.8	1.5	**91**	***59.3***	***39.5***	**135**	96.4	90.6
**48**	7.5	1.7	**92**	***61.4***	***41.6***	**136**	96.6	91.2
**49**	7.8	1.9	**93**	***62.4***	***42.6***	**137**	96.7	91.7
**50**	8.2	2.4	**94**	***63.3***	***43.9***	**138**	96.7	92.3
**51**	9.0	2.9	**95**	***64.8***	***45.6***	**139**	96.8	93.1
**52**	9.7	3.5	**96**	***65.8***	***47.4***	**140**	97.1	94.0
**53**	10.4	3.9	**97**	***66.4***	***48.4***	**141**	97.3	95.0
**54**	10.8	4.1	**98**	***67.2***	***49.4***	**142**	97.6	95.3
**55**	11.8	4.2	**99**	***67.9***	***50.4***	**143**	97.9	95.9
**56**	12.8	4.4	**100**	***68.9***	***51.8***	**144**	98.3	96.2
**57**	13.8	4.7	**101**	***70.2***	***53.8***	**145**	98.6	96.5
**58**	14.1	4.9	**102**	***71.4***	***55.3***	**146**	98.9	96.8
**59**	15.0	5.4	**103**	***72.2***	***56.6***	**147**	99.0	97.2
**60**	***16.3***	5.8	**104**	***73.6***	***57.2***	**148**	100.0	100.0

Note. Area of normality (between PR 15.8 and PR 84.2; i.e. M +/- SD) is printed in bold italics.

**Table 6:** QADS factors standard percentages for males (N = 1098) and females (N = 1251)

Raw value	Animal Reminder Disgust		Core Disgust		Contamination Disgust	
	Male	Female	Male	Female	Male	Female
**1**	--	--	1.4	.8	--	--
**2**	--	--	2.6	1.7	.2	--
**3**	--	--	4.2	2.1	.4	--
**4**	--	--	7.0	3.4	.6	--
**5**	--	--	9.3	4.0	1.0	.2
**6**	--	--	11.4	5.1	1.7	.3
**7**	.1	--	13.1	6.5	2.6	.5
**8**	.1	--	15.7	8.1	3.6	.8
**9**	.1	--	***18.5***	10.0	4.5	1.0
**10**	.2	--	***21.6***	11.8	6.4	1.1
**11**	.4	--	***24.6***	14.3	7.4	1.4
**12**	.7	.2	***28.5***	***16.9***	9.0	1.9
**13**	.8	.2	***31.5***	***19.3***	10.3	2.3
**14**	1.0	.3	***35.6***	***21.4***	11.8	3.1
**15**	1.4	.5	***38.7***	***24.2***	13.4	4.0
**16**	1.5	.6	***42.9***	***28.2***	15.3	5.1
**17**	1.8	.7	***47.3***	***32.1***	***18.1***	6.7
**18**	2.2	.7	***52.4***	***36.5***	***20.9***	8.2
**19**	3.1	.8	***56.5***	***40.8***	***24.4***	10.1
**20**	4.0	.9	***61.3***	***45.2***	***27.3***	11.9
**21**	4.2	1.2	***64.8***	***50.6***	***29.5***	13.4
**22**	4.8	1.6	***69.2***	***54.8***	***33.3***	15.7
**23**	5.6	1.9	***72.7***	***59.0***	***37.7***	***18.6***
**24**	6.0	2.4	***76.3***	***62.8***	***41.8***	***22.0***
**25**	6.5	2.6	***79.9***	***66.2***	***45.9***	***26.0***
**26**	7.3	3.2	***82.2***	***69.5***	***50.8***	***30.1***
**27**	8.7	4.0	***85.2***	***73.5***	***54.5***	***34.6***
**28**	10.7	4.6	87.6	***76.0***	***57.7***	***38.4***
**29**	12.9	5.8	90.2	***79.8***	***60.9***	***41.2***
**30**	15.6	6.8	91.9	***83.6***	***62.8***	***45.2***
**31**	***18.3***	8.5	93.3	86.1	***67.1***	***49.0***
**32**	***21.1***	10.5	95.1	89.0	***70.1***	***52.5***
**33**	***23.3***	11.9	96.1	91.0	***72.7***	***54.7***
**34**	***25.6***	14.3	97.1	93.2	***76.0***	***58.1***
**35**	***28.9***	***16.7***	98.3	95.0	***78.4***	***62.0***
**36**	***33.1***	***18.1***	100.0	100.0	***80.4***	***64.5***
**37**	***36.0***	***20.2***			***82.6***	***67.1***
**38**	***38.7***	***22.5***			***83.9***	***69.4***
**39**	***42.1***	***24.9***			86.0	***74.0***
**40**	***45.4***	***27.7***			87.4	***76.9***
**41**	***48.5***	***30.7***			89.3	***80.0***
**42**	***52.1***	***33.7***			91.3	***82.3***
**43**	***55.4***	***37.3***			92.7	84.8
**44**	***58.0***	***40.8***			93.5	86.8
**45**	***62.7***	***46.0***			94.7	88.9
**46**	***65.9***	***50.0***			95.4	90.3
**47**	***69.0***	***52.7***			96.1	92.0
**48**	***71.6***	***56.0***			97.1	93.6
**49**	***75.0***	***60.0***			97.4	94.8
**50**	***78.1***	***63.3***			98.2	96.0
**51**	***81.0***	***66.7***			98.6	96.8
**52**	***84.1***	***69.9***			100.0	100.0
**53**	86.7	***73.9***				
**54**	88.8	***78.3***				
**55**	90.9	***82.5***				
**56**	92.1	85.8				
**57**	94.3	88.6				
**58**	95.7	90.7				
**59**	97.3	93.2				
**60**	100.0	100.0				

Note. Area of normality (between PR 15.8 and PR 84.2; i.e. M +/- SD) is printed in bold italics.

## Discussion

The present study aims at analyzing the factor structure of the Questionnaire for the Assessment of Disgust Sensitivity (QADS), a generalized self-report instrument that assesses the individual disposition of 'disgust sensitivity'. For the first time, this analysis was based on a large representative sample.

The adjustment of the statistical procedures during the exploratory and confirmatory factor analyses to the non-normality of item distributions led to a model with three statistically coherent, data-driven, theoretically sound, and highly correlated factors. This model moderately fits the presented data from a representative German sample, and fits better than an unrestricted one-factor model or the five-factor model proposed by Schienle and colleagues [1].

Factor one of the preferred three-factor model assembles 15 items: all but one Oral Rejection item, five items of Body Secretions, three Spoilage items and two of Poor Hygiene. The idea behind this factor can be considered as *Core Disgust *according to Rozin and colleagues [3], i.e. disgust triggered by the threat of disease through mostly oral contact and a sense of offensiveness, including stimuli such as rotten food and body secretions.

Factor two consists of nine items: all the Death/Deformation items as well as two Spoilage items that overlap with Death as they point to dead creatures. This factor clearly reminds people of their animal origin and mortal nature as *Animal Reminder Disgust *suggests [3].

Factor three incorporates 13 items: seven Poor Hygiene items, three Spoilage items, two Body Secretions items and one Oral Rejection item. The major concept behind this factor can be referred to as *Contamination Disgust*, representing reactions toward the perceived threat of contagion through mainly non-oral contact, i.e. inhalation or skin contact (see [3,5] p.285).

It can be assumed that the three identified factors overlap mainly in content with the three factors of the Disgust Scale - Revised (DS-R) [5]. Although 15 QADS items (items 8, 10 to 15, 27 to 33, 35, 36) are also included in the 25 DS-R items, the QADS is more than a mere German DS translation. It contains several items that strongly contribute to the respective factor but are not included in the original DS, e.g. items 3, 13, 16, 19, 23, and 34. Future research could investigate a combined item pool of both the DS-R and the QADS while examining the convergent validity of these measurement instruments.

Item-scale-correlations were medium to high, replicating the findings by Olatunji and colleagues [5]. The QADS factors inherited very good reliabilities of .90 to .95, which were slightly higher than those reported by Schienle and colleagues [1] for the five original QADS factors (.69 to .85). They were also higher than those presented for the DS and DS-R with a reliability of .84 [4] and an internal consistency of < .70 [5], respectively. This might have been due to diverse item scaling in the DS and its revised version or too few items per factor in the original QADS.

The QADS factors were demonstrated as unaffected by the age of the participants. This might be due to the probably time-stable nature of the trait-like concept. Gender influences, however, were found. Women were moderately more sensitive to disgust than men. Similar gender effects were reported earlier [4,17] and are of importance as they mediate gender differences in disgust-related disorders, e.g. contamination fear in individuals with an obsessive compulsive disorder [18].

Construct validity on a conceptual level should be given as the items were partly derived from established disgust sensitivity measures, i.e. the DS and the DS-R. Construct validity on a factorial level is supported by recent findings by Olatunji and colleagues [19]. Convergent validity was also tested by van Overveld and colleagues ([20], see also [5]) using the *Disgust Propensity and Sensitivity Scale *by Cavanagh and Davey [21]. A comparison of QADS and DS-R to this or other generalized disgust instruments such as Wronska's questionnaire [22] and the *Disgust Emotion Scale *by Kleinknecht and colleagues [23] still need to be carried out in large representative samples and with adequate statistical methods adjusted to possibly non-normal item distributions.

Another aspect is that the concept of disgust sensitivity might need to be refined since some of the popular definitions rely on the empirical basis of now revised findings. The DS-R, for example, explicitly relies on the theoretical concepts by Rozin and colleagues [3] instead of several more or less independent domains as the DS did. The underlying three-factor model of disgust sensitivity is further supported by recent findings as all three factors were demonstrated to be distinct in relation to personality characteristics, behavioral, and physiological reactions as well as clinical fears [19]. Olatunji and colleagues [19] speak of "kinds of disgusts" while referring to what we call "disgust sensitivity", hereby not precisely distinguishing between the emotion of disgust and the underlying trait-like preparedness to experience disgust, Van Overveld and colleagues [20] postulate an even more sophisticated view on disgust sensitivity. They argue that the concept itself should be renamed *disgust propensity*, i.e. how easily a person becomes disgusted in opposition to the tendency of experiencing something as 'horrid', or rather how strongly a person is bothered by it, which they call *disgust sensitivity*. It seems important to note that the QADS factors are named according to the disgust sensitivity concept by Rozin and colleagues [3] since both the QADS items and DS-R items have not yet been divided into propensity items and sensitivity items.

## Conclusion

Developed as a paper-and-pencil test with as few as 37 five-point scaled items, the QADS can be applied easily and quickly. In sum, the QADS is age-independent, objective, reliable, and now standardized on a representative sample with tabulated standard percentages for both genders. On account of its advantageous design being comprised of three different factors, it can be used to assess specific disgust domains as well as dispositional disgust sensitivity via the Main Score. Limitations are due to unexplored overlaps with other disgust sensitivity measures, their items, and factor structures. To be specific, a comparison of the QADS, DS-R and other generalized disgust sensitivity instruments still needs to be conducted. For theoretical reasons, including "moral disgust" items could be considered [3].

## Competing interests

The authors declare that they have no competing interests.

## Authors' contributions

EB and CA were responsible for the conception and the design of the study as well as the acquisition of the data. GS and MR performed the statistical analysis and substantially contributed to the interpretation of the data. KP and SP contributed to the interpretation of the data, wrote the first and final version of the manuscript, and critically revised the manuscript for intellectual content.

All the authors read and approved the final version of the manuscript for publication.

## Pre-publication history

The pre-publication history for this paper can be accessed here:

http://www.biomedcentral.com/1471-2288/10/95/prepub

## Supplementary Material

Additional file 1 **Fragebogen zur Ekelempfindlichkeit (QADS)**. The original German questionnaire including the instruction. Click here for file
