# Supplementary material for: Domains of disgust sensitivity: revisited factor structure of the questionnaire for the assessment of disgust sensitivity (QADS) in a cross-sectional, representative german survey
Source: BMC Med Res Methodol. 2010 Oct 20;10:95. doi: 10.1186/1471-2288-10-95 (PMC2984502; doi:10.1186/1471-2288-10-95)
Supplement: Additional file 1 — Fragebogen zur Ekelempfindlichkeit (QADS). The original German questionnaire including the instruction. [file 1471-2288-10-95-S1.PDF]

# Fragebogen zur Ekelempfindlichkeit

**Im Folgenden werden verschiedene Situationen beschrieben. Bitte stufen Sie ein, wie eklig diese Situationen für Sie wären.**

| | | nicht eklig | ←→ | | | sehr eklig |
|---|---|---|---|---|---|---|
| 01 | **Sie beobachten, wie sich eine Person nach dem Gang zur Toilette nicht die Hände wäscht.** | ☐1 | ☐2 | ☐3 | ☐4 | ☐5 |
| 02 | **Sie beißen in eine gegrillte Heuschrecke.** | ☐1 | ☐2 | ☐3 | ☐4 | ☐5 |
| 03 | **Sie riechen Erbrochenes.** | ☐1 | ☐2 | ☐3 | ☐4 | ☐5 |
| 04 | **Sie entfernen eine tote haarige Spinne aus Ihrer Wohnung.** | ☐1 | ☐2 | ☐3 | ☐4 | ☐5 |
| 05 | **Im Bus setzt sich jemand neben Sie, der intensiv nach Schweiß riecht.** | ☐1 | ☐2 | ☐3 | ☐4 | ☐5 |
| 06 | **Sie gehen in eine Gruft, in der Särge stehen.** | ☐1 | ☐2 | ☐3 | ☐4 | ☐5 |
| 07 | **Sie essen ein Steak, bei dem das Innere noch blutig ist.** | ☐1 | ☐2 | ☐3 | ☐4 | ☐5 |
| 08 | **Sie probieren, Affenfleisch zu essen.** | ☐1 | ☐2 | ☐3 | ☐4 | ☐5 |
| 09 | **Ein Bekannter erzählt Ihnen, dass er grundsätzlich kein Deo verwendet.** | ☐1 | ☐2 | ☐3 | ☐4 | ☐5 |
| 10 | **Sie sehen eine Kakerlake im Haus eines anderen.** | ☐1 | ☐2 | ☐3 | ☐4 | ☐5 |
| 11 | **Sie hören, wie sich jemand mit Schleim im Rachen räuspert.** | ☐1 | ☐2 | ☐3 | ☐4 | ☐5 |
| 12 | **Sie beobachten wie sich jemand übergibt.** | ☐1 | ☐2 | ☐3 | ☐4 | ☐5 |
| 13 | **Sie berühren einen toten Körper.** | ☐1 | ☐2 | ☐3 | ☐4 | ☐5 |
| 14 | **Sie berühren mit einem Teil Ihres Körpers die Klobrille in einer öffentlichen Toilette.** | ☐1 | ☐2 | ☐3 | ☐4 | ☐5 |
| 15 | **Sie gehen in Ihr Lieblingsrestaurant und finden heraus, dass der Koch erkältet ist.** | ☐1 | ☐2 | ☐3 | ☐4 | ☐5 |
| 16 | **Sie fahren in einem Leichenwagen mit.** | ☐1 | ☐2 | ☐3 | ☐4 | ☐5 |
| 17 | **Sie haben einen Löffel Suppe gegessen, als Sie mit der Zunge ein Haar spüren** | ☐1 | ☐2 | ☐3 | ☐4 | ☐5 |
| 18 | **Sie holen einen Topf aus dem Kühlschrank und öffnen ihn. Der Geruch von verdorbenem Essen zieht Ihnen in die Nase.** | ☐1 | ☐2 | ☐3 | ☐4 | ☐5 |
| 19 | **Eine Person mit schmutzigen Fingernägeln reicht Ihnen ein Buch.** | ☐1 | ☐2 | ☐3 | ☐4 | ☐5 |
| 20 | **Während eines Waldspaziergangs sehen Sie einen verwesten Tierkadaver.** | ☐1 | ☐2 | ☐3 | ☐4 | ☐5 |
| 21 | **Als Unfallhelfer sollen Sie eine stark blutende Wunde abdrücken.** | ☐1 | ☐2 | ☐3 | ☐4 | ☐5 |
| 22 | **Ihnen zieht ein unangenehmer Geruch in die Nase. Sie blicken an sich herab und sehen, dass Sie in einen Hundehaufen getreten sind.** | ☐1 | ☐2 | ☐3 | ☐4 | ☐5 |

Fortsetzung auf der nächsten Seite ➔

## Fortsetzung Fragebogen zur Ekelempfindlichkeit

| | | nicht eklig ◄ | | | | ► sehr eklig |
|---|---|---|---|---|---|---|
| 23 | **Sie benutzen eine stark verschmutzte Autobahntoilette.** | ☐1 | ☐2 | ☐3 | ☐4 | ☐5 |
| 24 | **Sie fassen den Totenschädel eines Menschen an.** | ☐1 | ☐2 | ☐3 | ☐4 | ☐5 |
| 25 | **Eine Person mit starkem Mundgeruch spricht Sie an.** | ☐1 | ☐2 | ☐3 | ☐4 | ☐5 |
| 26 | **Sie haben versehentlich den Stumpf eines armamputierten Mannes berührt.** | ☐1 | ☐2 | ☐3 | ☐4 | ☐5 |
| 27 | **Sie sehen wie jemand Ketchup über ein Vanilleeis gießt und es isst.** | ☐1 | ☐2 | ☐3 | ☐4 | ☐5 |
| 28 | **Sie sind gerade dabei, ein Glas Milch zu trinken, als Sie riechen, dass sie verdorben ist.** | ☐1 | ☐2 | ☐3 | ☐4 | ☐5 |
| 29 | **Sie sehen Maden auf einem Stück Fleisch draußen in einem Mülleimer.** | ☐1 | ☐2 | ☐3 | ☐4 | ☐5 |
| 30 | **Sie laufen barfuß auf der Straße und treten auf einen Regenwurm.** | ☐1 | ☐2 | ☐3 | ☐4 | ☐5 |
| 31 | **Während Sie durch eine Bahnunterführung gehen, riechen Sie Urin.** | ☐1 | ☐2 | ☐3 | ☐4 | ☐5 |
| 32 | **Versehentlich berühren Sie die Asche einer Person, die eingeäschert wurde.** | ☐1 | ☐2 | ☐3 | ☐4 | ☐5 |
| 33 | **Sie sind hungrig. Vor Ihnen steht ein Teller Ihrer Lieblingssuppe, die mit einer benutzten, aber gründlich gewaschenen Fliegenklatsche gerührt wurde.** | ☐1 | ☐2 | ☐3 | ☐4 | ☐5 |
| 34 | **Sie sehen eine Person mit sehr fettigen Haaren.** | ☐1 | ☐2 | ☐3 | ☐4 | ☐5 |
| 35 | **In einem Restaurant sehen Sie jemanden, der unansehnliches Essen mit seinen Fingern isst.** | ☐1 | ☐2 | ☐3 | ☐4 | ☐5 |
| 36 | **Sie finden heraus, dass ein Freund von Ihnen nur einmal in der Woche seine Unterhosen wechselt.** | ☐1 | ☐2 | ☐3 | ☐4 | ☐5 |
| 37 | **Sie nehmen rohes Eiweiß in den Mund.** | ☐1 | ☐2 | ☐3 | ☐4 | ☐5 |

**Vielen Dank!**
